# Seroprevalence of Antibodies to Pertussis Toxin among Different Age Groups in Thailand after 37 Years of Universal Whole-Cell Pertussis Vaccination

**DOI:** 10.1371/journal.pone.0148338

**Published:** 2016-02-02

**Authors:** Nasamon Wanlapakorn, Varisara Ngaovithunvong, Thanunrat Thongmee, Preeyaporn Vichaiwattana, Sompong Vongpunsawad, Yong Poovorawan

**Affiliations:** Center of Excellence in Clinical Virology, Department of Pediatrics, Faculty of Medicine, Chulalongkorn University, Pathumwan, Bangkok, 10330, Thailand; Universidad Nacional de la Plata, ARGENTINA

## Abstract

Despite the high coverage of prophylactic vaccine against *Bordetella pertussis* infection in many countries for more than three decades, pertussis remains a common vaccine-preventable disease. Infections have been detected more commonly in countries using acellular pertussis vaccine in their Expanded Program of Immunization. Thailand implemented a routine infant immunization program with whole-cell pertussis vaccine in 1977, and since 1992, the national vaccine policy has offered a five-dose whole-cell pertussis vaccine for children given at the ages of 2, 4, 6, 18, and 48 months. This study aimed to investigate the seroprevalence of antibodies to pertussis toxin among healthy people across all ages to determine the level of whole-cell vaccine-induced immunity in the population, and to identify which age group should be targeted for a booster dose. The lowest seronegative rate and highest geometric mean concentrations were found in the 0–10 years age group, corresponding to their recent pertussis vaccination. The proportion of people with undetectable IgG level was prominent, starting after 11 years of age onwards. Now that a reduced-dose pertussis vaccine with fewer adverse effects is available, a booster dose during adolescence should be considered in order to reduce the incidence of pertussis disease. Further studies exploring how long the reduced-dose pertussis vaccine can provide protective immunity against pertussis disease when administered to adults and adolescents should also be performed.

## Introduction

Pertussis is a contagious bacterial disease of the respiratory tract characterized by a severe protracted paroxysmal cough that sometimes ends with a whooping sound, also known as “whooping cough.” It is caused mainly by *Bordetella pertussis* and is spread by direct contact with or breathing the aerosolized secretions of infected individuals. In adolescents and adults, the classic symptom of pertussis is a prolonged cough for 6–10 weeks, whereas in infants younger than 6 months, clinical manifestations are short catarrhal stage, gagging, cyanosis, apnea, or respiratory failure without whooping [1]. Despite the high coverage of prophylactic vaccine in many countries, *B*. *pertussis* infection remains a common vaccine-preventable disease and accounts for 139,786 cases reported worldwide in 2014 [2]. In the United States, the Centers for Disease Control and Prevention reported 28,660 cases and 9 deaths from pertussis in 2014, of which the majority (7/9; 77.8%) of the fatal cases were infants younger than 3 months of age [3].

Whole-cell pertussis (wP) vaccines consist of suspensions of the entire *B*. *pertussis* organism that has been inactivated. This type of vaccine was first licensed as part of routine infant vaccination in the United States in the mid-1940s, [4] and many countries have implemented this vaccine into their Expanded Program of Immunization (EPI). Although the vaccine was proven to be effective because the morbidity and mortality from pertussis disease decreased after its implementation in many countries, it was associated with several adverse local and systemic reactions, such as pain, redness and swelling at injection sites, high-grade fever, hypotonic–hyporesponsive episodes, and seizures [5]. In the 1980s, the acellular pertussis (aP) vaccine was introduced, which has also proven to be effective with fewer adverse effects. Therefore, many industrialized countries changed from the wP to the aP vaccine. However, head to head studies on the long-term efficacy of the wP and aP vaccines with regards to their efficacy in preventing the disease rather than their ability to induce antibody titers in vaccinees are limited.

Despite the high coverage of pertussis vaccine worldwide for many years, the incidence of pertussis has gradually increased. Recent research found that the waning of vaccine-induced pertussis immunity is one of the major contributing factors to pertussis disease [6,7]. Moreover, a woman who does not have protective anti-pertussis antibodies cannot pass the protection to her child during pregnancy, thus the newborn infant is susceptible to the disease. In this age group, pertussis may be severe and life-threatening [8].

Thailand implemented a routine infant immunization program with two doses of the diphtheria–tetanus toxoid–whole-cell pertussis (DTP) vaccine for all infants in 1977 [9], and this recommendation was changed to three doses of DTP in 1982 and four doses (at 2, 4, 6, and 18 months) in 1987. Since 1992, the national vaccine policy in Thailand has used five doses of DTP vaccine for children at the ages of 2, 4, 6, 18, and 48 months. Although many countries recommended a tetanus–diphtheria–acellular (Tdap) pertussis vaccine booster during adolescence [10–11], Thailand continued to use the whole-cell pertussis vaccine and integrated only the diphtheria–tetanus vaccine into its EPI for this age group and adults [12]. Although the number of pertussis cases from passive surveillance in Thailand was quite low between 2009 and 2014 (ranging from 0.01 to 0.11 cases per 100,000 population) with no report of major outbreaks [13–14], these numbers are likely to be underestimated because of inadequate disease surveillance and limited laboratory confirmation of suspected cases. The current laboratory diagnostic test for *B*. *pertussis* infection performed by The Ministry of Public Health for all hospitals in Thailand is the real-time polymerase chain reaction. In general, only specimens from clinically suspected cases or from a presumed outbreak would be collected and laboratory-tested.

To explore the serological profiles of anti-pertussis toxin IgG in a cohort of Thais who received only the wP vaccine in the past 37 years, we conducted a seroepidemiology survey to determine the antibody level across all ages. The results could help determine the level of wP vaccine-induced immunity and identify which age group should be targeted for a booster dose.

## Materials and Methods

The study was approved by the Institutional Review Board of the Faculty of Medicine, Chulalongkorn University (IRB No. 154/58), and was conducted in accordance with the principles of the Helsinki Declaration II. Permission for specimen utilization was granted by the Director of King Chulalongkorn Memorial Hospital. The sera analyzed in this study were collected between March and October 2014 for research on the impact of universal hepatitis B immunization of newborns as part of the EPI (IRB No. 419/56). All subjects had previously been informed about the pertussis study, and had provided written informed consent for use of their sera in this study. All information and patient identifiers were kept anonymous in order to strictly protect patient confidentiality.

### Seroprevalence of antibodies against pertussis toxin

In total, 900 serum samples were randomly selected from people residing in seven provinces from four geographical regions of Thailand: Uttaradit and Phitsanulok province in the north, Lop Buri and Ayutthaya province in central, Trang and Narathiwat province in the south, and Khon Kaen province in the northeast (S1 Fig). There were 252 samples from Khon Kaen and 108 samples from each of the other provinces. The samples were obtained from both male and female volunteers, and their ages ranged from 12 days to 64 years. The sera were stored at -20°C until tested.

### Laboratory methods

The presence of anti-pertussis toxin (anti-PT) IgG was detected by quantitative analysis using a commercially available ELISA kit (EUROIMMUN, Lübeck, Germany) and interpreted according to the manufacturer’s instructions. The lower limit of detection for the kit is 0.2 IU of anti-PT IgG per ml of serum. Level >100 IU/ml indicated acute pertussis infection or recent vaccination, while 40–100 IU/ml were interpreted as probable past exposure to pertussis. Level of 5–40 IU/ml was interpreted as no evidence of recent acute infection and <5 IU/ml indicated seronegativity.

### Pertussis vaccine coverage in Thailand and types of pertussis vaccine currently used worldwide

Data regarding pertussis vaccine coverage in Thailand were retrieved from the Department of Disease Control, Thai Ministry of Public Health. In addition, data on the types of pertussis vaccine used worldwide were retrieved from the online database of the World Health Organization [15].

### Statistical analysis

The IgG level was expressed as the geometric mean concentrations (GMC) with 95% confidence interval. For the calculation of the GMC, undetectable values were excluded. Antitoxin levels and data according to age group were analyzed using SPSS software (version 20; IBM Inc., Armonk, NY, USA). One-way analysis of variance was used to evaluate statistically significant differences in the GMC between different age groups. The χ^2^ test was used to compare the proportion of different antibody levels between individuals. Any differences were considered statistically significant at P<0.05.

## Results

### Seroprevalence of antibody against pertussis toxin

The 900 samples were divided into six age groups (0–10, 11–20, 21–30, 31–40, 41–50, and >50 years) (Table 1). The overall GMC of anti-PT antibody in the population was 5.83 IU/ml. The highest GMC was found in the 0–10 years age group (GMC 12.81 IU/ml, 95% CI = 10.30–15.91), and the lowest in the 31–40 years age group (GMC 3.76 IU/ml, 95% CI = 2.87–4.93). The only statistically significant differences were observed between the GMC from group 0–10 years and those from groups 21 years and older.

**Table 1:** Age-specific Geometric Mean Concentrations (GMC) of anti-pertussis toxin IgG in the Thai population.

Age (years)	n	GMC	95% CI	Of GMC	
			Lower	Upper	*p-value*
0–10	154	12.81	10.31	15.91	Reference
11–20	147	5.22	4.01	6.80	0.449
21–30	147	4.53	3.47	5.91	0.007*
31–40	153	3.76	2.87	4.93	0.004*
41–50	149	6.04	4.78	7.63	0.011*
>50	150	5.57	4.47	6.94	0.001*
Total	900	5.83	5.26	6.46	

* denote statistical significance.

When samples were classified according to the antibody level (<5 IU/ml, 5–40 IU/ml, 40–100 IU/ml, and >100 IU/ml), we found that the majority of the population had antibody levels of 5–40 IU/ml (48.5%), followed by < 5 IU/ml (42.1%) (Table 2). However, 23 samples (2.6%) had antibody levels >100 IU/ml indicating a recent infection or vaccination. Of these 23 samples, nine (39.1%) were aged between 6 months and 5 years, six (26%) were aged 11–16 years, and the remaining eight (34.9%) were aged >21 years. Among 61 subjects with antibody levels between 40 and 100 IU/ml, 18 (29.5%) were ages 0–10 years and 43 (70.5%) were ≥11 years-old.

**Table 2:** Anti-pertussis toxin IgG level in the Thai population.

Anti-pertussis toxin IgG (IU/ml)	n	Percent	95% CI	of Percent
		(%)	Lower	Upper
<5 (IU/ml)	379	42.1%	38.9	45.4
5–40 (IU/ml)	437	48.5%	45.2	51.9
40–100 (IU/ml)	61	6.8%	5.2	8.6
>100 (IU/ml)	23	2.6%	1.6	3.8

**Fig 1:**
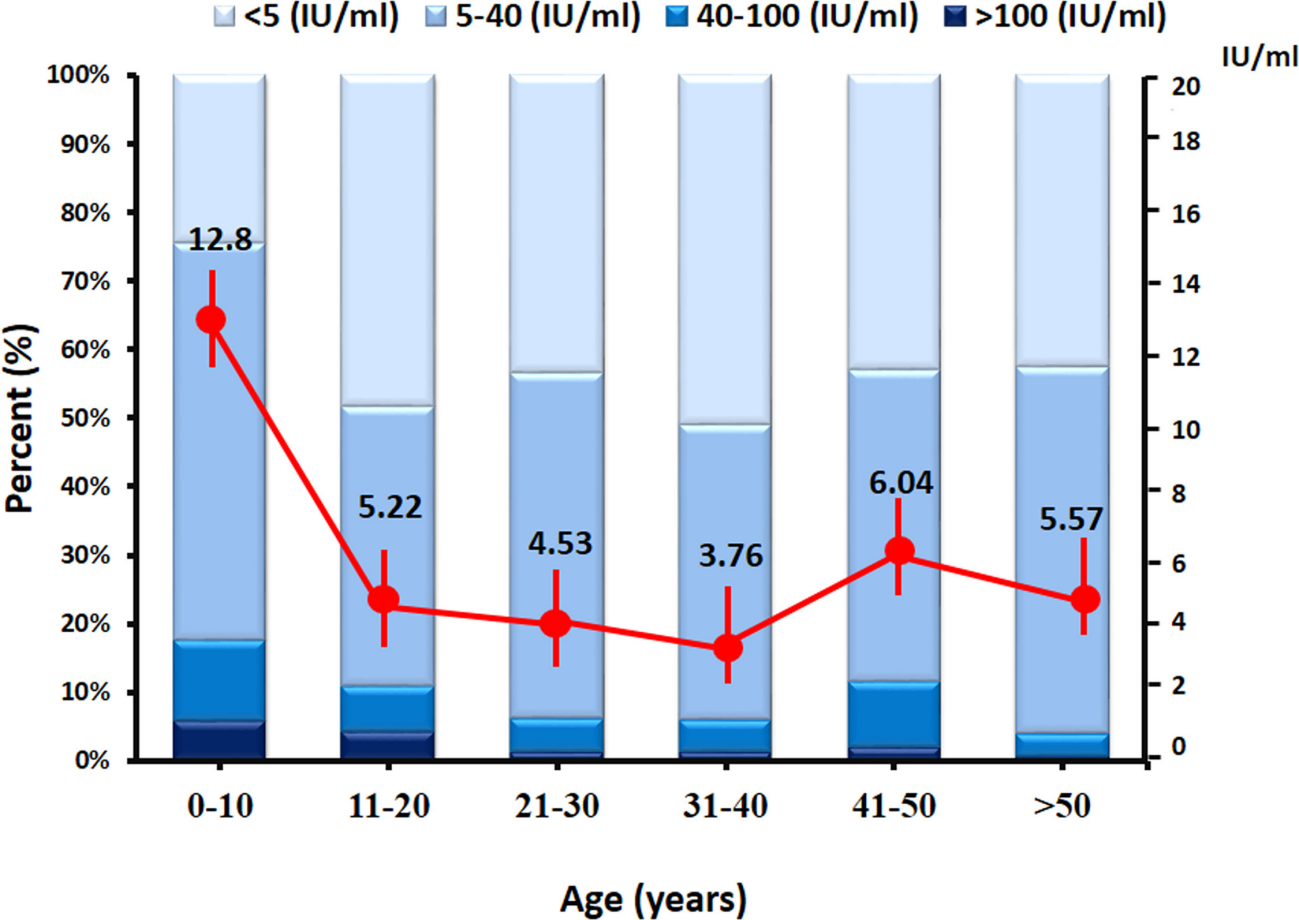
Age-specific anti-pertussis toxin antibody level and Geometric Mean Concentrations (GMC) in the Thai population in 2014. Six age groups were 0–10, 11–20, 21–30, 31–40, 41–50 and >50 years (x-axis). Scale on the left represented the percentage of population with different antibody levels. Scale on the right represented the GMC in each age group with the means indicated as red dots (red vertical lines denote 95% CI). Antibody measurements were <5 IU/ml (very light blue), 5–40 IU/ml (light blue), 40–100 IU/ml (blue) and >100 IU/ml (dark blue).

Comparison of the seropositivity rate by age group (Fig 1) revealed that children aged <10 years had the lowest proportion of seronegativity (24.7%), which was significantly different compared with the other age groups (P<0.01). The highest seronegativity rate was observed in the 31–40 years age group (51%), followed by the 11–20 years (48.3%), 21–30 years (43.5%), 41–50 years (43%), and >50 years (42.7%) age groups. The anti-PT IgG levels of the population residing in the seven provinces showed that the seronegativity rates were not statistically different among the provinces (S2 Fig). Individuals from Ayutthya province had the highest proportion of titers >100 IU/ml (5.6%), while the highest GMC were detected in people residing in Lop Buri province (8.25 IU/ml), followed by Trang province (7.11 IU/ml).

### Pertussis vaccine coverage in Thailand and vaccine types currently used worldwide

**Fig 2:**
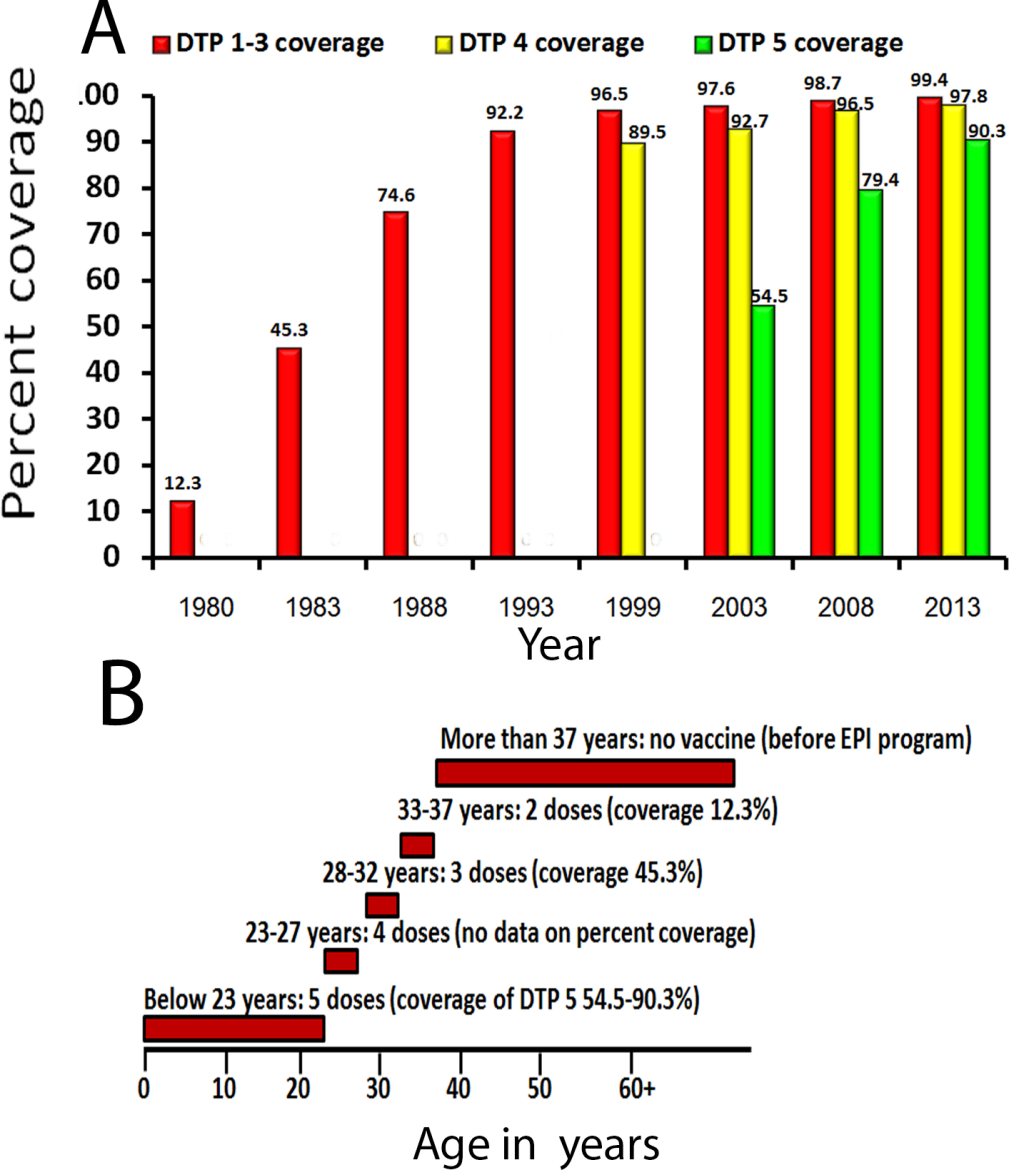
Coverage of diphtheria–tetanus and whole cell pertussis (DTP) vaccine in Thailand, 1980–2013. (A) Increasing vaccine coverage beginning in 1980. The red bar represented vaccination coverage for 3 completed doses (DTP 1–3) when administered to infants at 2, 4 and 6 months of age. The yellow bar represented coverage for 4 completed doses (DTP 4), which included an additional dose at 18 months. The green bar represented coverage for 5 completed doses (DTP 5), which included a fifth dose at 48 months. (B) The age at which individuals received the pertussis vaccine according to the Expanded Program of Immunization in Thailand. Data on percent coverage were summarized in parentheses.

In order to evaluate the whole-cell vaccine-induced immunity in the Thai population, we retrieved the data on vaccine coverage surveyed every five years from the Department of Disease Control, Thai Ministry of Public Health. We found that more than 95% of Thai children have received three doses of pertussis vaccine since 1999 (Fig 2). These children are now adolescents at the time of the seroprevalence survey. The coverage of the fourth dose of pertussis vaccine reached > 95% in 2008, meaning that children under six years of age in this survey have received at least four doses. However, coverage with five doses was much harder to achieve and did not reach 95% according to the latest survey in 2013. As of 2014, half of the countries worldwide use aP vaccine in their EPI, but many countries in Asia, Africa and South America still rely on wP vaccine in their universal vaccination programs [15].

## Discussion

To our knowledge, this study is the first population-based cross-sectional seroepidemiology survey of anti-PT in the Thai population. Thailand implemented a wP vaccine program for infants in 1977, and since then, it has not begun using aP vaccine as in the USA and other countries in Europe. The current EPI in Thailand gives no booster dose of pertussis vaccine to adults or adolescents. Therefore, the purpose of the current study was to compare the patterns of anti-pertussis toxin IgG across all ages from both the pre- and post-vaccination eras, and to identify the appropriate time for a booster dose. Overall, the lowest seronegative rate and the highest GMC were found in the 0–10 years age group, corresponding to the most recent pertussis vaccinations. The proportion of undetected IgG level was prominent from 11 years of age onwards. Our study found that the seronegative rate was 48.3% among adolescents aged 11–20 years who had received at least three to four doses during childhood, indicating that antibody titers from vaccine-induced immunity do not last long even with the wP vaccines. In addition, among older individuals who were born before the EPI or those who received fewer than three doses of pertussis vaccine, the seronegative rate was similar to adolescents. These observations suggest that even in the pre-vaccination era when the population was exposed to *B*. *pertussis*, antibody levels did not remain positive and high throughout the lifetime.

Serosurveys in countries using wP vaccines have also reported waning of immunity. A study in Iran involving 640 students aged 6–17 years showed that 53% were seronegative and susceptible to pertussis despite five-dose wP vaccination at 2, 4, 6, 18 months and 4–6 years [16]. A study in Pakistan demonstrated that 77.4% of children 10–12 years-old who were vaccinated with wP vaccine at 6, 10, and 14 months of age were seronegative [17]. Despite the low level of antibody to pertussis toxin within ten years after vaccination, the clinical efficacy against pertussis disease with wP vaccination was as high as 92% when evaluated at six years after the last dose [18]. Although the results of antibody concentration after vaccination and clinical efficacy have been somewhat conflicting, the Advisory Committee on Immunization Practices (AICP) recommends a booster dose of the Tdap vaccine during adolescence, regardless of the type of pertussis vaccine received during infancy, in order to increase antibody level and provide better protection against pertussis [19]. The proportion of seronegative individuals in this study was approximately 43–51% after 11 years of age, which may indicate susceptibility to pertussis infection in these individuals. The result of this study provided evidence of low pertussis antibody concentration in the Thai population. Therefore, inclusion of the Tdap vaccine into the Thai EPI program for adolescents according to the recommendations from the AICP should be considered.

Our study demonstrated that there were 23 samples with >100 IU/ml of anti-PT IgG. Of these 23 participants, 9 were aged between 6 months and 5 years, indicating the effect of childhood vaccination, while the remaining 14 participants were aged above 11 years. As we did not have accurate vaccination data for our study population, we could not make a strong conclusion as to whether the high pertussis titers in our study resulted from recent vaccination or recent exposure to pertussis. However, due to the very low coverage of Tdap vaccination among Thai adolescents and adults and the last doses of pertussis vaccination according to the Thai EPI were given before six years of age, it is possible that 14 participants may have been exposed to pertussis, resulting in the high antibody titers. In addition, among 61 participants whose antibody concentrations were between 40–100 IU/ml, 43 were aged ≥11 years and likely represented exposure to pertussis in the past years. If we used the cut-off point of > 40 IU/ml to calculate the number of individuals likely exposed to pertussis, around 6.3% (57 in 900) of Thais aged above 11 years likely had pertussis infection in the past years.

This result is comparable with a seroepidemiological study conducted in Denmark [20]. Using a cut-off point of > 50 IU/ml, the prevalence of pertussis infection in the population was 5.6% (95% CI 4.1–7.8). In the Netherlands, one study found that 9.3% of the population aged > 9 years had antibody level of more than 62.5 EU/ml, suggestive of pertussis infection in previous years [21]. An estimate of the incidence of pertussis based on serological titers in five European countries found that the seroincidence of infection was approximately 1–6% per annum with a peak in adolescent to young adult age group [22]. China also reported that approximately 5.2% of adult population 18–50 years of age had pertussis infection using a cut-off point of antibody level > 30 IU/ml [11]. However, a recent study emphasized that the incidence estimates of pertussis infection based on antibody measurement alone were higher than the incidence from the annual report [23]. Nevertheless, passive surveillance of the incidence of pertussis in Thailand could have underestimated the actual incidence. Despite the high coverage of wP vaccine for many years, pertussis continued to circulate in the Thai population.

There were some limitations to this study. No information regarding participants’ immunization status was available, therefore it was difficult to confirm the cause of the high antibody levels found. In addition, cut-off points to define seropositive samples in pertussis seroprevalence studies differ depending on the enzyme-linked immunosorbent assay kit. This could affect the percentages of seropositive individuals when compared to other studies. With the availability of a reduced-dose pertussis vaccine, which induces fewer adverse effects, a booster dose during adolescence should be considered for better protection against pertussis disease among the Thai population. Further studies should also be conducted to explore how long the reduced-dose pertussis vaccine administered to adults and adolescents can provide protective immunity.

## Supporting Information

S1 Fig The map of Thailand showing the seven provinces from which serum samples were taken. Uttaradit and Phitsanulok represent northern provinces; Lop Buri and Ayutthaya represent central provinces; Trang and Narathiwat represent southern provinces; and Khon Kaen represents a northeastern province (all denoted in red).
(TIF)

S2 Fig Anti-pertussis toxin IgG levels by province. Proportions of the population with different antibody levels (denoted in percent) ranged from <5 IU/ml (very light blue), 5–40 IU/ml (light blue), 40–100 IU/ml (blue) and > 100 IU/ml (dark blue).
(TIF)
