# Supplementary figures and images for: Seroprevalence of Antibodies to Pertussis Toxin among Different Age Groups in Thailand after 37 Years of Universal Whole-Cell Pertussis Vaccination
Source: PLoS One. 2016 Feb 2;11(2):e0148338. doi: 10.1371/journal.pone.0148338 (PMC4737491; doi:10.1371/journal.pone.0148338)

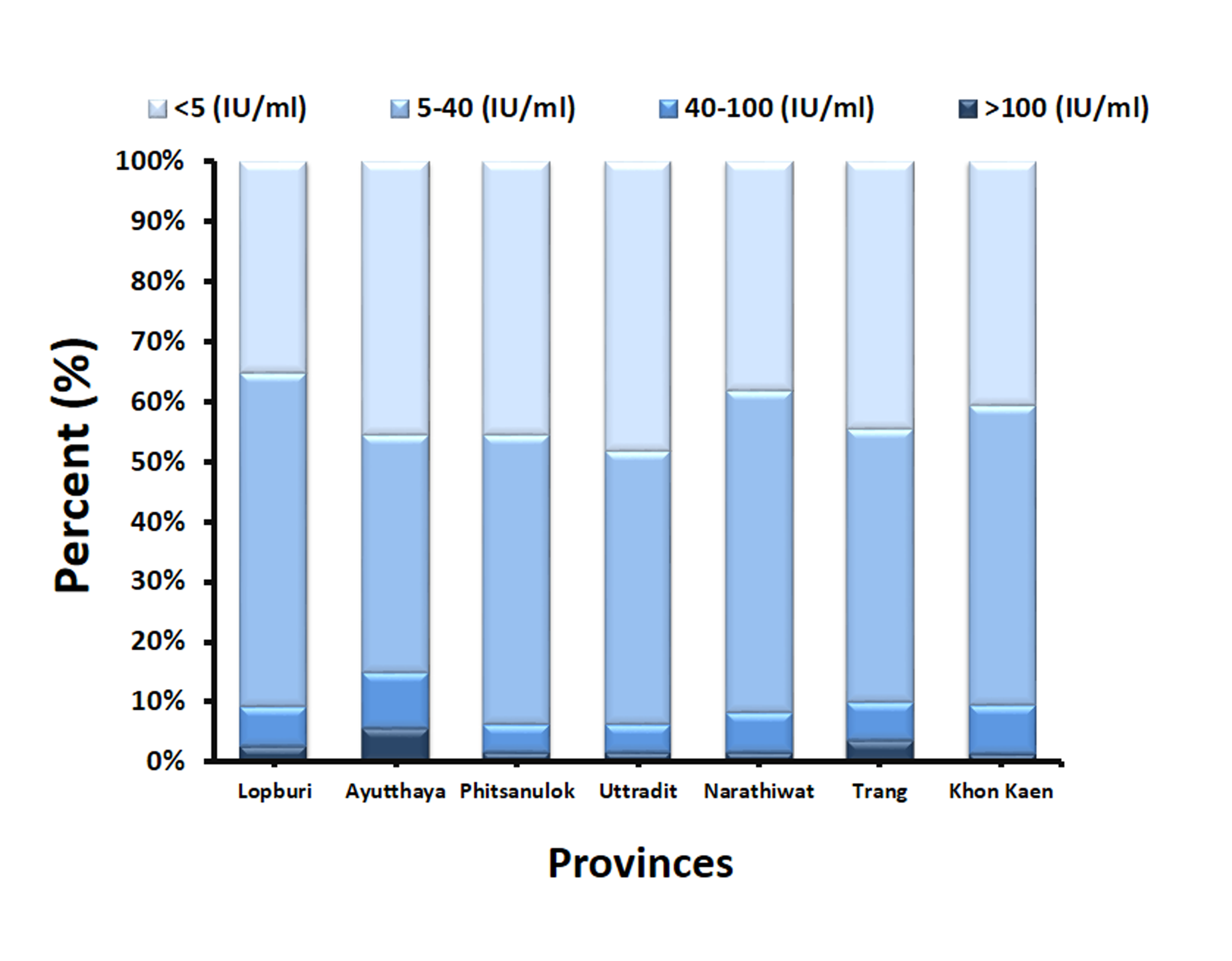

Supplement: S2 Fig — Proportions of the population with different antibody levels (denoted in percent) ranged from <5 IU/ml (very light blue), 5–40 IU/ml (light blue), 40–100 IU/ml (blue) and > 100 IU/ml (dark blue). (TIF) [file pone.0148338.s002.tif]
